# Compensation Claims After Treatment of Achilles Tendon Ruptures in Norway From 2010 to 2020

**DOI:** 10.1177/24730114251361475

**Published:** 2025-08-20

**Authors:** Tor Kristian Molstad Andresen, Per-Henrik Randsborg, Ståle Bergman Myhrvold, Rune Bruhn Jakobsen

**Affiliations:** 1 Department of Orthopaedic Surgery, Akershus University Hospital, Lørenskog, Norway; 2 Institute of Clinical Medicine, Faculty of Medicine, University of Oslo, Oslo, Norway

**Keywords:** Achilles tendon, compensation claims, patient injury

## Abstract

**Background:**

Compensation claims caused by medical treatment errors provide valuable insights into patients' experiences with received medical treatment and can help identify injuries and disabilities that might be preventable through optimized care. The objective of this retrospective descriptive study was to characterize accepted compensation claims filed after treatment for acute Achilles tendon rupture (ATR) in Norway, and to explore whether claim outcomes were associated with treatment modality or institutional catchment area.

**Methods:**

All claims filed to the Norwegian System of Patient Injury Compensation (NPE) after treatment of ATR between 2010 and 2020 were collected and categorized. The claims were organised based on whether they were accepted or denied, the initial treatment given, patient demographics, the reasons given by patients for filing claims, and the rationale provided by the NPE for accepting or rejecting compensation claims. Additionally, hospital patient catchment population were analyzed in relation to accepted claims.

**Results:**

One hundred forty-six compensation claims were received, of which 61 (41.8%) were accepted. Most accepted claims were related to surgical treatment (n = 30, 49%) or insufficient treatment (n = 22, 36%). The most frequent reason for claim acceptance was delayed diagnosis and/or treatment (15/61, 25%), followed by postoperative infections (10/61, 16%). There was no statistically significant correlation between accepted claims and institutional catchment area population.

**Conclusion:**

Delayed or missed diagnosis was the most common reason for accepted compensation claims following treatment for ATR. The study found no correlation between accepted claims and the catchment area population of the institution delivering the treatment.

**Level of Evidence:** Level IV, retrospective cohort study.

## Introduction

Achilles tendon ruptures (ATRs) commonly occur during sports activities, primarily affecting individuals in their working years. Both the mean age and incidence have been increasing over time, with a mean age of around 50 years and incidence estimated at approximately 64 ATRs per 100 000 persons annually for men and around 19 ATRs per 100 000 persons annually for women in Scandinavia.^26^ ATR can lead to severe disability, both in the short and the long term.^9^ Untreated or neglected ATRs will result in poor function, and late surgical repair and reconstruction yield a higher complication risk.^15^ ATR can be treated nonoperatively—typically with a cast and subsequent orthosis—or surgically with primary repair of the tendon and subsequent protection with cast and orthosis. There is no gold standard treatment, and the different treatment options have different complication profiles.^13,17^

Patient injury is defined as an injury caused by the treatment, or lack of such, provided by the health care system.^12^ Since 1988, claims for compensation after patient injuries in Norway are handled through the Norwegian System of Patient Injury Compensation (NPE).^5^ NPE is a government-run “no-blame” compensation scheme funded through obligatory payments from all institutions providing health care, both public and private. Patients file their claim for compensation by submitting an online form, at no cost for the patient and generally without legal aid. The task of the NPE is to grant compensation if a patient injury has occurred during treatment based on a no-blame principle assessed by a neutral expert with access to the patient’s journal and claim without jurisdictional involvement. The expert is trained in compensation regulations, and in advanced cases more than one reviewer is assigned. The claim is processed and either accepted or rejected. The decision is based on the principle of avoidability, meaning that the injury would most likely not have happened if treatment had adhered to current practice guidelines. This system of no-blame separates the handling of compensations claims from issues of legal malpractice. Therefore, most indemnity cases in Norway are settled outside the judicial system as some reasons for patient injuries are covered by the given exemption clause.

Three main conditions must be met to qualify for compensation. First, the patient must have suffered an injury caused by the examination, diagnosis, treatment (or lack of treatment), or follow-up. This means that the health care provided must be considered erroneous or substandard by the NPE. Second, the patient injury must either have resulted in a financial loss, currently set at a minimum of 10 000 NOK (approximately 850 EUR), or the treatment error must have resulted in permanent medical disability of at least 15%. Third, the compensation claim must be filed within 20 years and no more than 3 years after the patient recognized that he or she may have been subject to a patient injury. All claims are archived and available for analysis in anonymized form.

The purpose of the present retrospective, descriptive study was to review the compensation claims reported to the NPE following treatment of acute ATR and assess any correlations between treatment modality, reasons for compensation claim, and hospital catchment area population.

## Material and Methods

Data from all claims filed to the NPE regarding patient injury due to treatment error following ATR (*International Classification of Diseases, Tenth Revision*, code S86.0) from 2010 through 2020 were collected from the NPE. The data were retrospectively categorized and analyzed by the authors. We recorded the gender and age of the patients at time of injury, the type of primary treatment, the patient’s reason for filing the claim, the type of treatment injury, and NPE’s reason for accepting or rejecting the claim (Tables 1-3). Treatment error would be an error in the diagnosis, treatment, or follow-up (or lack thereof) that led to an injury that the medical experts deemed would not have occurred if treatment had adhered to current guidelines.^7^

**Table 1.** Patient Demographics and the Primary Treatment of 146 Compensations Claims for Patient Injury Following ATR in Norway Between 2010 and 2020.

Demographics	Accepted Claims (n = 61)	Rejected Claims (n = 85)	Total (N = 146)
Age, y, mean (range)	48 (18-77)	47 (20-81)	47.5 (18-81)
Women, n (%)	13 (21)	31 (37)	44 (30)
Primary treatment			
Surgical, n (%)	30 (49.2)	40 (47.1)	70 (48.2)
No treatment, n (%)	22 (36.1)	11 (12.9)	33 (24.5)
Nonoperative, n (%)	8 (13.1)	27 (31.8)	35 (22.5)
Unknown, n (%)	1 (1.6)	7 (8.2)	8 (4.9)

Abbreviation: ATR, Achilles tendon rupture.

**Table 2.** Patient-Reported Reasons for Filing a Claim to the NPE During 2010-2020 and Reasons for Accepted (Right Top) or Rejected Claims After Treatment for ATR.

Reason for Filing Claim		Reason for Accepted and Rejected Claims	
	n (%)		n (%)
Total claims	146	Reason for accepted claim	61
Functionally inferior result	37 (25.3)	Delayed diagnosis and treatment	16 (26.2)
Delayed diagnosis	34 (23.3)	Prolonged recovery	7 (11.5)
Infection	29 (19.9)	Infection led to multiple procedures	7 (11.5)
Inferior result due to nonoperative treatment	9 (6.2)	Inadequate examination	6 (9.8)
Pain	9 (6.2)	Inadequate follow-up	5 (8.2)
Deep vein thrombosis	5 (3.4)	Wrong treatment	5 (8.2)
Inadequate follow-up	4 (2.7)	Not referred to specialist	4 (6.6)
Prolonged recovery	3 (2.1)	Infection	4 (6.6)
Delayed surgery	3 (2.1)	ATR related to corticosteroid injection	2 (3.3)
Re-rupture	3 (2.1)	Tight cast	2 (3.3)
CRPS	2 (1.4)	Inadequate antithrombotic prophylaxis	1 (1.6)
Pulmonary embolism	2 (1.4)	Nerve injury perioperatively	1 (1.6)
Wrong cast	2 (1.4)	Wrong rehabilitation	1 (1.6)
Adhesions	1 (0.7)		
ATR related to corticosteroid injection	1 (0.7)	Reason for rejected claim	85
Inadequate information	1 (0.7)	No treatment error	40 (47.1)
Nerve injury	1 (0.7)	No correlation between complaint and treatment	28 (32.9)
		No failure in diagnosis	14 (16.5)
		Infection due to patient factors	3 (3.5)

Abbreviations: ATR, Achilles tendon rupture; CRPS, complex regional pain syndrome; NPE, Norwegian System of Patient Injury Compensation.

**Table 3.** Reason for Claim According to the Patients (Main Headings), With Reasons for Compensation Given by NPE Listed Below Each Subcategory.

Infection	22
Infection lead to multiple procedures	7
Prolonged recovery	7
Wrong treatment	3
Delayed diagnosis and treatment	2
Inadequate examination	1
Inadequate follow-up	1
Infection	1
Delayed diagnosis	15
Delayed diagnosis and treatment	6
Inadequate examination	4
Inadequate follow-up	3
Not referred to specialist	2
Functionally inferior result	13
Delayed diagnosis and treatment	4
Infection	3
ATR related to corticosteroid injection	2
Not referred to specialist	2
Inadequate examination	1
Wrong treatment	1
Prolonged recovery and/or pain	3
Delayed diagnosis and treatment	3
Thromboembolism	2
Inadequate antithrombotic prophylaxis	1
Inadequate follow-up	1
CRPS	2
Nerve injury perioperatively	1
Tight cast	1
Delayed surgery	1
Wrong treatment	1
Inadequate follow-up	1
Delayed diagnosis and treatment	1
Re-rupture	1
Wrong rehabilitation	1
Nerve injury	1
Tight cast	1
Total	61

Abbreviations: ATR, Achilles tendon rupture; CRPS, complex regional pain syndrome; NPE, Norwegian System of Patient Injury Compensation.

**Figure 1.**
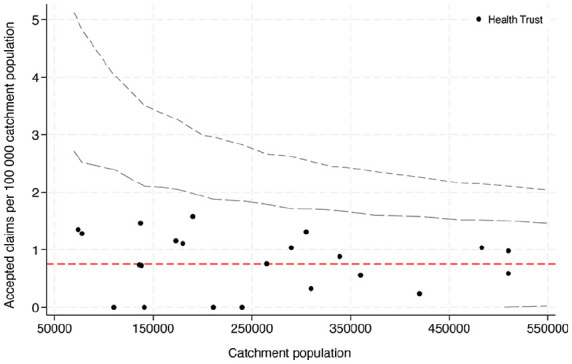
Funnel plot showing the number of accepted claims per 100 000 catchment population with control limits at 95% and 99.8% (corresponding to 2 SD and 3 SD from the mean [red line]).

The public clinics and hospitals in Norway are organised into 4 health trusts, each responsible for its unique geographical region. The catchment area population varies between the different health trusts, depending on the size of the region and the population density. In this public health system, care for most acute injuries (including ATR) is handled by the local hospital, determined by the patient’s home address. The data were analyzed for any correlation between accepted claims and hospital catchment area population (Figure 1).

### Statistical Methods

Patient demographics were analyzed and are presented by descriptive statistics. Continuous variables were described by means and SDs, whereas categorical data were reported in frequencies. We compared the rate of accepted claims per 100 000 catchment area volume between health trusts using funnel plot with control limits set at 95% and 99.8%.^24,28^ The analyses were conducted in Statistical Package for the Social Sciences (SPSS, version 27.0; IBM Corp, Armonk, NY) and Stata 18.0 SE (StataCorp LLC, TX).

## Results

During the study period, 146 compensation claims related to ATR were filed to the NPE, of which 61 (42%) were accepted. Approximately two-thirds of the claims were filed by men, with a mean age of 47.5 years (Table 1).

The largest proportion of filed claims, both accepted and rejected, were associated with surgical treatment (49% and 47% respectively) followed by claims filed by patients who did not receive any primary treatment because of delayed or missed diagnosis (24%) (Table 1).

The most frequent reason given by the patient for filing a claim was inferior functional outcome (25.3%), followed by delayed diagnosis (23.3%) and postoperative infection (19.2%) (Table 2). The most frequent reason for accepted claims as reported by the NPE was delayed diagnosis and treatment (Table 2).

By comparing the patient’s reason for filing a compensation claim with the NPE’s reason for acceptance, we illustrate how patients’ experiences and reviewers’ assessments of avoidability sometimes diverged (Table 3).

The number of patients with accepted claims was identical for nonoperatively and surgically treated patients, with 30 cases in each group (Table 4). For 1 patient who experienced a rupture after a corticosteroid injection the treatment of the ruptured tendon was not specified and hence categorized as unknown. Of those treated non-operatively half of the accepted claims (15 of 30) were due to delayed diagnosis and/or treatment. Except from 4 cases, all accepted claims from those treated nonoperatively were due to missed early diagnosis, delayed treatment and/or lack of follow-up. Postoperative infection (10 of 30) was the most frequent reason for accepted claim for those treated surgically, and 6 of these underwent surgical revision. Of the 61 accepted claims, 25 involved individuals aged 50 years or older, of these, 11 (44%) had undergone surgical treatment. In the rejected group, 37 claims were recorded, with 13 individuals (35%) receiving surgical treatment.

**Table 4.** Reasons for Accepted Claims After Treatment for ATR filed to the NPE During 2010–2020 Categorized by Treatment Modality.

Nonoperative Treatment		Surgical Treatment	
	n		n
Delayed diagnosis and/or treatment	15	Infection	10
Inadequate examination	5	Prolonged recovery	7
Not referred to specialist	3	Wrong treatment	3
Inadequate follow-up	2	Inadequate follow-up	3
ATR related to corticosteroid injection	1	Tight cast	1
Infection led to multiple procedures	1	Not referred to specialist	1
Tight cast	1	No indication for surgery	1
Wrong rehabilitation	1	Nerve injury perioperatively	1
Wrong treatment	1	Inadequate examination	1
Unknown		Inadequate antithrombotic prophylaxis	1
ATR related to corticosteroid injection	1	Delayed diagnosis and treatment	1

Abbreviations: ATR, Achilles tendon rupture; NPE, Norwegian System of Patient Injury Compensation.

There was no statistically significant difference in the proportion of accepted compensation claims following ATR between the different hospitals when analyzed according to their catchment area population (Figure 1).

## Discussion

This study identifies the most common causes of patient injury during the diagnosis, treatment, and follow-up following acute ATR in Norway. By acknowledging these errors, we provide lessons for future caregivers. However, these findings do not reflect all complications of ATR treatment, but rather the subset that led to accepted claims and met the NPE's thresholds for compensation. The most frequently cited reasons for accepted compensation were delayed diagnosis and/or treatment followed by prolonged rehabilitation and postoperative infections. The different reasons for filing a compensation claim when comparing nonoperative and surgical treatment reflect the different adverse risk profiles between the treatment modalities. Major adverse events, such as re-rupture following nonoperative treatment and deep infection following surgical treatment, are often associated with inferior outcomes, with or without the need for additional surgical procedures. Inferior outcomes after major adverse events were reflected in the accepted compensation claims among surgically treated patients in our study, with infection being the largest group.^2,16^ Only 1 nerve injury was reported among all claims, despite prospective studies indicating an incidence of 1% to 7%, with the majority occurring after surgical treatment of ATR.^6,17^ This discrepancy may reflect that, although troublesome, most sensory nerve injuries following both surgical and, to a lesser extent, nonoperative treatment are transient and self-limiting.^19^ Additionally, such injuries may be considered a recognized surgical risk that does not necessarily indicate a treatment error. Of the filed claims due to pain, only 2 were granted. Both were described as iatrogenic nerve injury with persisting pain, classified as complex regional pain syndrome. However, and perhaps surprisingly, only 3 claims (2%) were filed because of re-rupture, and only 1 of these claims was accepted because of too aggressive rehabilitation. A re-rupture is a well-known complication that may occur without a treatment error, and patients inferring a re-rupture are therefore not entitled to compensation for this complication alone if the treatment and follow-up adheres to recommended guidelines.

The exploration of compensation claims has garnered increased interests as an adjuvant in understanding the course of health care provided for different ilnesses.^1,20^ Sveen and coworkers described 324 acute ATR registered in the Danish system of patient injury compensation over an 18-year long period, of which 129 (40%) was accepted, which is similar to our finding of 42%.^27^ The authors discovered a 3.8 times higher compensation rate after surgical treatment of ATR compared with nonoperative treatment. Similar to the findings in our study, the most frequent reason for compensation was an overlooked primary diagnosis (34.5%). The importance of avoiding missed or incorrect diagnosis was also a major finding among compensations claims in the United Kingdom following femoral neck fractures.^23^ Among the accepted claims, we identified infections as the second most common factor, with 11 cases (18%). This suggests that early diagnosis, appropriate intervention, and nonoperative management could potentially reduce the number of accepted claims. Our finding of 42% acceptance of the filed claims were in line with other studies on compensation claims after orthopaedic injuries.^4,10,21^

There is no consensus to as which form of treatment leads to superior outcome in acute Achilles tendon ruptures.^17^ But delayed treatment, or no treatment at all, is associated with longer recovery and may contribute to worse outcomes.^25^ It is therefore not surprising that a majority of the claims after nonoperative treatment was compensated because of delayed diagnosis and treatment. In the case of chronic ruptures, both the process of treatment and recovery is more costly compared with early diagnosis and intervention.^18^ In our categorization, “no treatment” does not indicate nonoperative treatment, but rather neglected, missed diagnosis or delayed treatment possibly causing patient injury. In cases where the patient caused the delay, the claim was rejected. Similar finding has been described for hip fractures and anterior cruciate ligament ruptures.^21,23^

The incidence of missed primary diagnosis and/or neglect by the patient as a reason for late contact with the health care is not clear, as patient-delay would not itself be regarded as a treatment error and hence is not included in this study. Earlier publications based on case reports and retrospective studies have estimated the proportion of late presentation to be as high as 25%, although this is most likely an overstatement given the increased awareness and tools for diagnosis.^11^ The diagnosis of ATR is based on clinical examination usually provided by primary health care and junior doctors in the health trusts’ outpatient clinics. In the case of uncertainty in the diagnosis, the patient might be neglected or referred to additional examinations such as ultrasonography, magnetic resonance imaging, or a specialist. Treatment delayed due to waiting time for radiologic examination, particularly magnetic resonance imaging, which is both time-consuming and costly, is another observed reason for filing a complaint.^8^ Although it might add certainty to the diagnosis, radiologic examinations are not necessary to diagnose the majority of acute ATRs. A clinical diagnosis can be established with an excellent accuracy by the cluster of tests often referred to as the Simmonds triad: palpable gap in the tendon, altered angle of declination (increased dorsiflexed position of the foot of the injured leg with the patient in prone position), and a positive Thompson test (no plantarflexion when calf muscles are squeezed) with the addition of decreased plantigrade force.^3,14,22^ Of the rejected claims, 11 patients received no active treatment. NPE contends that these rejections stemmed from insufficient causal evidence, potentially because of patient-related factors such as delayed medical consultation.

In contradiction to compensation studies on other orthopaedic conditions, we did not see a correlation to the hospital catchment volume.^4,10^ This may be explained by the relative simplicity of the diagnosis and surgical procedure, as well as the relatively low number of claims in our study. We estimate the annual incidence of acute ATR in Norway to be approximately 1800. This yields a 0.8% incidence of filing a claim following treatment for ATR, which is similar to the incidence of claims reported after hip fracture surgery (0.7%) and slightly lower than the rates determined in the Danish ATR compensation study (1.2%).

### Limitations

This study has some limitations. The study is from a single country with a no-blame compensation scheme, which is different from compensation schemes found in other countries. This may limit the external validity. However, the purpose of the study was to analyze the causes and aspects of accepted compensation claims because of treatment errors after treatment of Achilles tendon rupture, and we have no reason to believe that the treatment of Achilles tendon rupture in Norway differs significantly from other comparable countries. As such, the results should be of interest also outside of Norway. Furthermore, it is important to point out that this is not a study on treatment modality or its complications. Most complications will never be reported to NPE. A study on compensation claims due to treatment injuries evaluates the health care provided, not the clinical outcome of the treatment in question. Some patients who have suffered a treatment error may never report it to the NPE. Moreover, the reliance on accepted claims may introduce selection bias, as these cases represent a small, nonrandom subset of all ATR treatments and complications.This may influence our results, especially if the culture of reporting treatment errors or informing the patients about the right to file a claim differ between small and large health trusts.

## Conclusions

Delayed or missed diagnosis was the most common reason for accepted compensation claims after treatment of Achilles tendon rupture.

We saw no correlation between accepted claims and the catchment volume of the institution delivering the treatment.

## Supplemental Material

sj-pdf-1-fao-10.1177_24730114251361475 – Supplemental material for Compensation Claims After Treatment of Achilles Tendon Ruptures in Norway From 2010 to 2020 Supplemental material, sj-pdf-1-fao-10.1177_24730114251361475 for Compensation Claims After Treatment of Achilles Tendon Ruptures in Norway From 2010 to 2020 by Tor Kristian Molstad Andresen, Per-Henrik Randsborg, Ståle Bergman Myhrvold and Rune Bruhn Jakobsen in Foot & Ankle Orthopaedics
